# Comparison of Two Multidisciplinary Occupational Rehabilitation Programs Based on Multimodal Cognitive Behavior Therapy on Self-Rated Health and Work Ability

**DOI:** 10.3389/fpsyg.2021.669770

**Published:** 2021-08-23

**Authors:** Peter Solvoll Lyby, Thomas Johansen, Per M. Aslaksen

**Affiliations:** ^1^CatoSenteret Rehabilitation Center, Son, Norway; ^2^Norwegian National Advisory Unit on Occupational Rehabilitation, Rauland, Norway; ^3^Department of Psychology, UiT The Arctic University of Norway, Tromsø, Norway

**Keywords:** occupational rehabilitation, return to work, multidisciplinary rehabilitation, multimodal cognitive behavior therapy, musculoskeletal pain, common mental disorders

## Abstract

**Objective:** Musculoskeletal pain and common mental disorders constitute the largest proportion of people who are on sick leave. This study investigated the efficacy of two multidisciplinary occupational rehabilitation programs on self-rated health and work-related outcomes. The interventions were identical in content but differed in length. It was hypothesized that a longer inpatient program would yield greater improvements than a shorter outpatient program.

**Methods:** Patients were sick-listed workers referred to occupational rehabilitation by the Norwegian Labor and Welfare Administration. A non-randomized 2 Condition (20 days, *n* = 64 versus 12 days, *n* = 62) × 4 repeated measures (start, end, 3 months, 12 months) between-subject design was used. Both programs were based on multimodal cognitive behavior therapy with a return-to-work focus. Health-related questionnaires were the Subjective Health Complaints inventory, Hospital Anxiety and Depression Scale, and SF-36 Bodily Pain. Work-related questionnaires were the Work Ability Index, the Fear-Avoidance Beliefs Questionnaire, Return To Work Self-Efficacy, and Return To Work expectations. Intervention effects were estimated using linear mixed models and Cohen’s d.

**Results:** The results revealed that both groups improved on the selected outcomes. Within-group contrasts and effect sizes showed that the inpatient group showed larger effect sizes at the end of rehabilitation and 12 months post-intervention for work-related outcomes than the outpatient group.

**Conclusion:** Both programs were efficacious in improving health- and work-related outcomes during and after rehabilitation, but the inpatient group generally displayed stronger and more rapid improvements and was more stable at one-year postintervention.

## Introduction

Chronic musculoskeletal pain and common mental disorders (CMDs) are the two most frequent causes of disability and long-term sick leave in Western countries. In Norway, CMD, such as depression and anxiety, accounts for approximately one-fifth of all sickness absences, whereas musculoskeletal pain, such as back and neck pain, accounts for nearly half of all sickness absences (Brage et al., 2010). Additionally, research has long revealed extensive comorbidity of psychological and somatic symptoms in patients seeking occupational rehabilitation (Barnett et al., 2012; Brendbekken et al., 2017; Gismervik et al., 2020).

Norwegian occupational rehabilitation programs have had a biopsychosocial and transdiagnostic profile for nearly two decades in which patients with both chronic pain and CMD have been attending the same rehabilitation programs (Aasdahl et al., 2018). Typical treatment components are physical exercise, relaxation training, cognitive behavioral therapy (CBT) components for CMD and pain management, and work-related problem solving (Fimland et al., 2014). Such multidisciplinary rehabilitation programs (MRPs) have been developed to improve health and work ability and accelerate return to work (RTW).

Due to the multicausality and high-level clinical heterogeneity embedded in transdiagnostic populations, MRPs are generally recommended for this patient population. However, MRPs are resource demanding, and there has been a need to explore other options. This has resulted in research efforts in which studies contrast MRPs with other active but less extensive interventions. However, to our knowledge, these studies have almost exclusively ended up comparing the effect of two independent variables in which both intervention content and length differ (Jensen et al., 2011, 2012; Roche-Leboucher et al., 2011; Brendbekken et al., 2016, 2017, 2018; Aasdahl et al., 2017, 2018; Salomonsson et al., 2017; Gismervik et al., 2020). This leaves an important issue unanswered because such designs fail to address what the optimal length or dosage of MRPs might be in relation to perceived health and RTW. To investigate the hypothesis of optimal length, one would have to rely on a design that compares two interventions with the same content but with different lengths. To our knowledge, no studies have explored this issue within musculoskeletal pain or transdiagnostic populations. This would represent new and important information for the field and for stakeholders and decision-makers.

In the present study, inpatient MRP was compared to shorter outpatient MRP (20 vs 12 days). Both programs were performed within four consecutive weeks at the same rehabilitation institution and by the same team of professionals (i.e., shared clinical context). The programs were based on multimodal CBT aimed at improving health and work-related processes to facilitate RTW. The study aimed to evaluate the clinical utility of the programs in improving reported health and work ability and to elaborate on the clinical meaningfulness of the results. We hypothesized that the inpatient program would demonstrate larger amounts of change in outcomes and display treatment effects of larger magnitude than the outpatient program.

## Materials and Methods

### Participants

Patients were diagnosed and referred to occupational rehabilitation by hospitals, general practitioners, or employment specialists at The Norwegian Labour and Welfare Administration (NLWA). The study included 126 patients who were either on partial or full sick leave. Sixty-four patients participated in the inpatient program, and 62 patients participated in the outpatient program (Table 1). The majority of patients in the study had an ICD-10 referral diagnosis in either the categories F, mental or behavioral disorders or M, diseases of the musculoskeletal system or connective tissue. All patients were referred for rehabilitation from their homes. Referral to the inpatient or outpatient program was done independently of health status and was based on the preference (i.e., cost, geographical proximity to the center) of the two NLWA—offices that had ongoing contracts with the rehabilitation center. The contracts were achieved through open competition with other private rehabilitation institutions and with NLWA as an employer organization.

**TABLE 1:** Baseline characteristics and referral diagnostics of participants.

**Variable**	**Inpatient program (*n* = 64)**	**Outpatient program (*n* = 62)**
Age mean (SD)	43.4 (9.1)	44.8 (9.1)
Women *n* (%)	36 (56)	41 (66)
Higher education *n* (%)	26 (41)	17 (27)
**Self-reported work status *n* (%)**		
No work	35 (55)	40 (69)
Full or part time	29 (45)	18 (31)
Self-reported sick leave status mean months at T1 (SD)	6.2 (3.6)	7.4 (3.8)
**Main diagnoses according to ICD-10**		
F. Mental and behavioral disorders	30	23
M. Diseases in the musculoskeletal system and connective system	13	26
Z. Factors influencing health status and contact with health services	6	7
R. Symptoms, signs, and abnormal clinical and laboratory findings, not elsewhere classified	1	1
G. Diseases of the nervous system	4	3
I. Diseases of the circulatory system	5	1
J. Disesases of the respiratory system	0	1
S. Injury, poisoning and certain other consequences of external causes	1	0
C. Malignant neoplasms	3	0
E. Endocrine, nutritional and metabolic diseases	1	0

The present study was part of a larger multicenter study with a primary purpose to investigate cognitive function in relation to occupational rehabilitation (Johansen et al., 2019). Inclusion was based on consecutive recruitment of participants aged between 18 and 67 and with the ability to understand the questionnaires and the instructions given by the examiner for each cognitive test. Exclusion criteria were history of head injury or having applied for disability pension (inclusion and exclusion criteria were the same as in Johansen et al., 2016). The study was approved by the South-East Regional Committee for Medical and Health Research Ethics (2013/1559). All participants provided written informed consent, and all procedures were conducted according to the Helsinki declaration.

### Design

A non-randomized 2 Group (inpatient, 20 days versus outpatient, 12 days) × 4 repeated measures (start, end, 3 months, 12 months) between subject design. The study was designed to analyze between-group differences (inpatient vs outpatient programs) and treatment effects within groups (amount and magnitude of change across time points).

### Intervention

The inpatient program lasted for 5 + 5 + 5 + 5 days across 4 weeks, whereas the outpatient program lasted for 3 + 3 + 3 + 3 days across 4 weeks. The interventions for both groups were provided in a shared clinical context. Both inpatients and outpatients were a part of the same clinical cohort during their rehabilitation stay. They attended group activities together, were met by the same team of professionals, and received the same kind of assessments at entry. The same rehabilitation components were present in both programs but to a lesser extent in the outpatient program. Thus, the difference between groups was the length of the interventions and that the outpatients commuted back to their home each day.

At entry, assessments of the patient’s work ability, physical fitness, current work situation, and health situation were carried out to tailor rehabilitation efforts. The treatment was given mainly as group activities and partially as individual consultations with therapists. Key interventions at the group level were physical activity, relaxation training, CBT components for CMD and pain management, and education sessions (both health and work-related). The work consultants carried out a structured interview examining employment/unemployment status, physical and mental strains at the workplace, and economic status for each patient. They followed up with interventions targeting work-related processes such as work-related problem solving and conducted telephone conferences with NLWA coordinators and/or workplace contact persons.

The patients were followed up by a multidisciplinary team including at least four of the following professionals: physician, physiotherapist, psychologist, work consultant, nurse/psychiatric nurse, and sports pedagogue. The multidisciplinary team had received certified training in CBT by CBT therapists from the Norwegian Association of CBT. In addition, the work consultants had attended workshops and training by the Norwegian National Advisory Unit on Occupational Rehabilitation in how to supervise work-related processes and intervene with work-related problem-solving.

### Norwegian Sickness Insurance

Individuals who are unable to work due to illness or injury are entitled to sick leave benefits from the Norwegian sickness insurance scheme for a maximum of 52 weeks. For the first 16 days, full compensation is provided by the employer and thereafter by the tax-paid national insurance system. If the individual is still unable to resume partial or full-time work after 1 year, a work ability assessment determines if further benefits for up to 3 years may be granted. The benefits after the first year are normally two-thirds of the wages the individual had prior to sick leave. The benefits can be combined with partial work resumption.

### Materials

#### Health-Related Questionnaires (Table 2)

The Subjective Health Complaints (SHC) inventory (Eriksen et al., 1999) was used to assess participants’ health complaints during the last 30 days according to pseudoneurology (e.g., sleep problems, tiredness, dizziness, anxiety, sad/depression; range 0–21) and musculoskeletal pain (e.g., neck pain, pain in the upper part of the back, pain in the lower part of the back, pain in arms, pain in shoulders; range 0–24). The items were measured using a four-point Likert scale from 0 = “not affected” to 3 = “seriously affected.” The Fear Avoidance Beliefs Questionnaire (FABQ; Waddell et al., 1993) was used to assess fear-avoidance beliefs for physical activity (range 0–24) and work (range 0–42) using a 7-point Likert scale from 0 = “completely disagree” to 6 = “completely agree”). Items seven and eight from the Short Form 36 Health Survey (SF-36; Ware and Sherbourne, 1992) were used to assess pain (range 1–6; 6-point Likert scale from “no pain to “very strong pain”) and pain interference with work (range 1–5; 5-point Likert scale from “not at all” to “extremely much”). The Hospital Anxiety and Depression Scale (HADS; Zigmond and Snaith, 1983) covered symptoms of anxiety (range 0–21) and depression (range 0–21) and was measured using a 4-point Likert scale (with differing endpoint formulations).

**TABLE 2:** Health-related outcomes: pre- and post-test scores (M, CI), within-group effect sizes (Cohen’s d with CI), and between-group effect sizes (Cohen’s d).

		**Inpatient (*N* = 64)**			**Outpatient (*N* = 62)**			**Between group d’s**
	**Time**	**Mean**	**95% CI**	**d (95% CI)**	**Mean**	**95% CI**	**d (95% CI)**	
**SHC**								
Pseudoneurology	Start intervention	6.9	5.8–8.0		6.5	5.4–7.6		
Range 0–21	End intervention	4.9***	4.0–5.9	0.80 (0.50–1.1)	5.6	4.5–6.6	0.38 (0.08–0.67)	0.42
	3 months	5.4**	4.3–6.4	0.54 (0.25–0.82)	5*	3.8–6.2	0.57 (0.23–0.91)	–0.03
	12 months	6	4.8–7.3	0.32 (0.03–0.61)	6	4.6–7.4	0.15 (−0.19 to 0.48)	0.17
Musculoskeletal pain	Start intervention	10.1	8.9–11.3		8.9	7.7–10.1		
Range 0–24	End intervention	7.4***	6.3–8.5	0.76 (0.47–1.1)	7.9**	6.8–9.1	0.30 (0.01–0.59)	0.46
	3 months	7.8***	6.6–9.0	0.52 (0.24–0.81)	6.7*	5.4–8.0	0.60 (0.25–0.95)	–0.08
	12 months	7.4***	6.2–8.6	0.62 (0.31–0.93)	7	5.6–8.4	0.56 (0.2–0.91)	0.06
**HADS**								
Anxiety	Start intervention	8.3	7.0–9.5		9.2	7.9–1.5		
Range 0–21	End intervention	7*	5.8–8.2	0.44 (0.17–0.71)	8.4	7.1–9.7	0.27 (−0.02 to 0.56)	0.17
	3 months	6.9*	5.7–8.0	0.41 (0.13–0.68)	7.4**	6.1–8.7	0.58 (0.24–0.92)	–0.17
	12 months	6.4**	5.1–7.7	0.70 (0.38–1.0)	7.1**	5.6–8.6	0.41 (0.05–0.75)	0.29
Depression	Start intervention	6.2	5.1–7.3		6.8	5.7–8.0		
Range 0–21	End intervention	4.3**	3.2–5.4	0.61 (0.33–0.89)	5.9	4.7–7.0	0.45 (0.15–0.74)	0.16
	3 months	5*	4.0–6.0	0.50 (0.22–0.78)	5**	3.9–6.2	0.68 (0.33–1.0)	–0.18
	12 months	4.6*	3.4–5.8	0.54 (0.23–0.85)	5.4	4.0–6.8	0.51 (0.15–0.86)	0.03
**SF-36**								
Pain	Start intervention	4.6	4.3–4.9		4.8	4.5–5.0		
Range 1–6	End intervention	4.2	3.9–4.5	0.34 (0.06–0.61)	3.9***	3.6–4.3	0.75 (0.44–1.06)	–0.41
	3 months	4.1**	3.8–4.4	0.56 (0.25–0.86)	3.8***	3.5–4.1	0.66 (0.34–0.98)	–0.10
	12 months	4.1*	3.7–4.4	0.50 (0.19–0.82)	3.8***	3.4–4.1	0.67 (0.32–1.0)	–0.17
Pain related to work	Start intervention	3.6	3.3–3.9		3.7	3.4–4.0		
Range 1–5	End intervention	2.9***	2.5–3.2	0.72 (0.42–1.0)	2.7***	2.4–3.0	0.93 (0.60–1.26)	–0.19
	3 months	3**	2.7–3.3	0.61 (0.29–0.92)	2.7***	2.4–3.0	0.91 (0.56–1.24)	–0.30
	12 months	2.8**	2.4–3.2	0.63 (0.31–0.96)	2.7***	2.3–3.0	0.68 (0.32–1.0)	–0.05

M, mean; CI, confidence interval; d, Cohen’s d. M and CI from linear mixed model (adjusted values). Cohen’s d based on raw scores are presented with CI’s. Between group d’s by subtracting outpatient d from inpatient d, positive numbers are in favor of the inpatient group, and negative numbers are in favor of the outpatient group. *p < 0.05; **p < 0.01; ***p < 0.001; indicating statistical significance for linear mixed model within group comparisions (start of intervention against the three post-tests).

#### Work-Related Questionnaires (Table 3)

Work ability was assessed using one item from the work ability index comparing current work ability with the lifetime best and measured on a 10-point numerical scale (0 = “no ability to work”, 10 = “my best work ability”) (Ahlstrom et al., 2010). Return to work self-efficacy (RTWSE-19; Shaw et al., 2011; Nottingnes et al., 2019) assesses the participants’ belief in their own ability to resume normal work tasks according to the following factors: meeting job demands, modifying job tasks, and communicating needs to others. The items were measured using ten-point numerical rating scales (1 = “not sure” to 10 = “completely sure”). RTW expectation was utilized by a one-item question asking about when the participant expected to RTW (based on Nielsen et al., 2011). Scale point definitions were 1 = “within first two weeks”; 2 = “within one month”; 3 = “within two months”; 4 = “within three months”; 5 = “within six months”; 6 = “within one year”; and 7 = “more than one year”.

**TABLE 3:** Work-related outcomes: pre- and post-test scores (M, CI), within-group effect sizes (Cohen’s d with CI), and between-group effect sizes (Cohen’s d).

		**Inpatient (*N* = 64)**			**Outpatient (*N* = 62)**			**Between group d’s**
	**Time**	**Mean**	**95% CI**	**d (95% CI)**	**Mean**	**95% CI**	**d (95% CI)**	
**Work ability**	Start intervention	3.8	3.2–4.5		3.5	28–4.2		
Range 0–10	End intervention	5.6***	4.9–6.3	0.91 (0.61–1.2)	4.5**	3.8–5.3	0.48 (0.17–0.77)	0.43
	3 months	5.4***	4.8–6.0	0.90 (0.59–1.2)	4.9***	4.2–5.7	0.94 (0.56–1.32)	–0.03
	12 months	5.7***	4.8–6.6	0.73 (0.41–1)	5.4***	4.4–6.4	0.69 (0.32–1.06)	0.04
**FABQ**								
Work	Start intervention	19.6	16.7–22.5		24.3	21.2–27.3		
Range 0–42	End intervention	18	15.1–21.0	0.33 (0.05–0.61)	22.2	19.0–25.5	0.29 (−0.02 to 0.59)	0.04
	3 months	16.9	14.0–20.0	0.41 (0.12–0.70)	20.4*	17.0–23.8	0.57 (0.20–0.93)	–0.16
	12 months	15.2*	11.4–19.1	0.53 (0.20–0.86)	22.6	18.3–26.8	0.13 (−0.22 to 0.48)	0.40
Physical activity	Start intervention	10.4	8.7–12.1		9.6	7.8–11.4		
Range 0–24	End intervention	7.6**	5.8–9.4	0.54 (0.25–0.82)	8.7	6.7–10.7	0.12 (−0.18 to 0.42)	0.42
	3 months	7.3***	5.7–9.0	0.66 (0.35–0.96)	7.9	6.0–9.7	0.44 (0.08–0.79)	0.22
	12 months	6.9***	5.1–8.8	0.73 (0.39–1.1)	10	7.9–12.0	−0.17 (−0.51 to 0.17)	0.90
**RTWSE-19**								
Meeting work demands	Start intervention	32.3	27.3–37.3		32.2	26.8–37.6		
Range 1–70	End intervention	40.3***	35.6–45.0	0.56 (0.28–0.85)	36.9	31.7–42.1	0.38 (0.06–0.69)	0.18
	3 months	43.3***	37.6–49.0	0.64 (0.33–0.95)	40.6*	33.7–47.5	0.58 (0.21–0.96)	0.06
	12 months	47***	39.8–54.1	0.76 (0.41–1.1)	40.7	32.3–49.0	0.45 (0.05–0.85)	0.31
Modyfying work tasks	Start intervention	28.9	25.1–32.6		28	24.0–31.9		
Range 1–60	End intervention	35**	31.7–38.4	0.49 (0.19–0.78)	32.5	28.7–36.3	0.36 (0.04–0.68)	0.13
	3 months	34.8*	30.8–38.7	0.50 (0.17–0.81)	32.2	27.5–36.9	0.51 (0.13–0.88)	–0.01
	12 months	36.4*	31.2–41.6	0.62 (0.25–0.98)	32.2	26.2–38.1	0.22 (−0.17 to 0.60)	0.40
Communicating needs	Start intervention	37	33.1–40.8		41.5	37.4–45.6		
Range 1–60	End intervention	40.4	36.7–44.2	0.28 (0.00–0.55)	40.1	36.0–44.2	−0.10 (−0.41 to 0.20)	0.38
	3 months	39.4	35.6–43.2	0.22 (0.07–0.51)	42.4	38.0–46.8	0.11 (−0.23 to 0.45)	0.11
	12 months	42	37.3–46.7	0.42 (0.09–0.76)	40.2	34.1–46.2	−0.14 (−0.52 to 0.24)	0.56
**RTW-expectations**	Start intervention	3.4	2.9–3.9		4.1	3.6–4.6		
Range 1–7^a^	End intervention	2.9*	2.4–3.3	0.40 (0.14–0.66)	3.4**	2.9–3.9	0.58 (0.26–0.89)	–0.18

M, mean; CI, confidence interval; d, Cohen’s d. M and CI from linear mixed model (adjusted values). Cohen’d based on raw scores are presented with CI’s. Between group d’s by subtracting outpatient d from inpatient d, positive numbers are in favor of the inpatient group, and negative numbers are in favor of the outpatient group. *p < 0.05; **p < 0.01; ***p < 0.001; indicating statistical significance for linear mixed model within group comparisions (Start of intervention against the three posttests). ^*a*^RTW-expectations; 1, within first two weeks; 2, within one month; 3, within two months; 4, within three months; 5, within six months; 6, within one year; 7, more than one year.

### Statistical Analysis

Group comparisons of outcome measures at baseline were analyzed by *t*-tests. *P*-values from the *t*-tests were adjusted for multiple comparisons by the Holm-Bonferroni procedure to control the type I error rate and reduce the risk of type II errors.

Repeated measures data were analyzed with linear mixed models (LMMs). The distribution of data for all dependent variables was inspected with Q-Q- and Boxplots, and data were deemed suitable for linear modeling. The models consisted of Group, Time, and Diagnosis (F = “mental”, M = “musculoskeletal”, Other) as factors. Gender (male, female), age in years at T1, and education level (0 = not completed primary school, 1 = completed primary school, 2 = completed high school, 3 = bachelor’s degree or lower at university/college, 4 = master’s degree or higher) were entered as covariates. We assumed that individual differences would impact the results, and therefore, we used the participants’ individual variance as a random factor with a random intercept in the models. The fixed-effect model consisted of the main effect of all the included variables and the Group by Time, Group by Diagnosis, and Group by Time by Diagnosis interactions. Significant effects of time and interactions were followed up with pairwise comparisons with Bonferroni adjustment of p-values for multiple comparisons.

Significant group-by-time interactions indicate meaningful between-group differences in contrasts over time (Start, End, 3, and 12 months are described as T1, T2, T3, and T4, respectively, in the result section). Within-group contrasts reflect the amount of change in outcomes over time within rehabilitation programs. Within-group effect sizes (Cohens *d*) were computed on raw scores (M, SD) and according to paired sample t-tests comparing T1 with T2–T4. Between-group differences in effect sizes were calculated by subtracting the within-group effect sizes from each other (Morris and Deshon, 2002; Frazier et al., 2015). The main and interaction effects of factors are presented in the text, and LMM estimates (M, CI) and d’s with CI are presented in Tables 2, 3. *P*-values <0.05 were considered significant for all analyses. Precision of the estimated means was assessed using 95% confidence intervals (CIs).

## Results

### Baseline

An overview of the demographics and baseline characteristics of the patients is presented in Table 1. *T*-tests showed that there were no significant differences between the two groups on any of the outcome measures, all *p* = 1, except for fear-avoidance work (*p* = 0.39) and RTW expectation (*p* = 0.39).

### Linear Mixed Model

The LMMs showed that the individual variance associated with each participant influenced the results. For all models shown below, the intercept for each participant showed significant variability with Wald Z’s > 4.1 and *p* < 0.01 for all analyses.

### Main Findings

**FIGURE 1:**
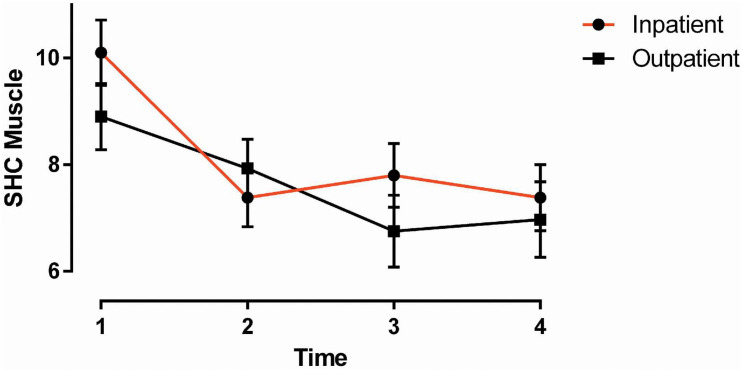
SHC Musculoskeletal Pain: group by time. Vertical bars denote 1 S.E.M.

We hypothesized that the inpatient group would demonstrate larger amounts of improvements and display treatment effects of larger magnitude. This can be evidenced by significant group-by-time interactions or, alternatively, by assessing the amount and magnitude of improvements across time points within groups. Consistent with our hypothesis, we found that the inpatient group improved more over time on musculoskeletal pain (Figure 1) and fear-avoidance for physical activity than the outpatient group. The other group-by-time interaction effects were non-significant.

Furthermore, Tables 2, 3 provide a complete overview of within-group contrasts and within- and between-group effect sizes. These results show that the inpatient group displayed significant improvements in 10 out of 13 outcome measures at the end of rehabilitation compared to 5 out of 13 in the outpatient group. The groups performed more similarly at 3 and 12 months postintervention. The frequency distribution of effect sizes (ES) according to Cohen’s categorization was as follows for the inpatient group contrasts: Zero ES (<0.2) = 0, Small ES (0.2) = 10, Medium ES (0.5) = 23, Large ES (0.8) = 5. For the outpatient group, the frequency distribution was zero ES (<0.2) = 7, small ES (0.2) = 12, medium ES (0.5) = 15, and large ES (0.8) = 3. The frequency distribution of between-group effect sizes larger than 0.2 (i.e., Small ES) were 12 in favor of the inpatient group. Three of these were found in contrasts for health-related outcomes. Two between-group effect sizes were in favor of the outpatient group, and both of these were found in the SF36 pain outcomes. In sum, within-group contrasts (*p*-values) and within- and between-group effect sizes provide evidence consistent with the hypothesis. The differences between groups were most frequently related to improvements from Start until End of the intervention, most frequently related to work-related outcomes, and the inpatient group performed on average with effect sizes with larger magnitudes.

### Between- and Within-Group Effects for Outcome Measures

#### SHC Pseudoneurological Symptoms

There was a significant main effect of time [*F* (3, 105.694) = 10.013, *p* ≤ 0.001]. Compared to pseudoneurological symptoms at T1, there were significant symptom reductions at T2 and T3 (*p* ≤ 0.001) and a non-significant reduction at T4 (*p* = 0.599). In the within-group comparisons, the inpatient group showed significant symptom reductions at T2 (*p* ≤ 0.001) and T3 (*p* = 0.008) but not at T4 (*p* = 0.785). In the outpatient group, there was a significant reduction in symptoms at T3 (*p* = 0.025). No symptom reductions were found at T2 and T4 (*p* = 0.140, 1.000).

There was an interaction effect of time by diagnosis [*F* (6, 105.659) = 2.215, *p* = 0.047], with pairwise comparisons showing a difference between the mental and musculoskeletal groups at T1 (*p* = 0.013).

#### SHC Musculoskeletal Pain

There was a significant interaction of Group by Time [*F* (3, 104.581) = 2.719, *p* = 0.048] (Figure 1), with the inpatient group showing slightly stronger reductions in musculoskeletal pain over time than the outpatient group. Between-group comparisons showed no significant differences at the respective time points. In the within-group comparisons, the inpatient group showed significant reductions in musculoskeletal pain at T2–T4 compared to T1 (all *p* ≤ 0.001). In the outpatient group, there was a significant symptom reduction at T3 and T4 compared to T1 (*p* = 0.004, 0.02).

There was a main effect of diagnosis [*F* (2, 107.679) = 3.49, *p* = 0.034], showing higher SHC in the musculoskeletal pain group than in the other group.

### Anxiety (HADS)

There was a significant main effect of time [*F* (3, 96.546) = 10.180, *p* ≤ 0.001]. Compared to T1, there were successive reductions in anxiety at T2–T4 (*p* ≤ 0.001, <0.001, 0.001). In the within-group comparisons, the inpatient group showed significant symptom reductions at all time points postintervention (*p* = 0.037, 0.014, 0.004). In the outpatient group, there was a non-significant symptom reduction at T2 (*p* = 0.635) and significant symptom reductions at T3 (*p* = 0.003) and T4 (*p* = 0.006).

### Depression (HADS)

There was a significant main effect of time [*F* (3, 93.784) = 10.796, *p* ≤ 0.001]. Compared to T1, there were successive reductions in depression at T2–T4 (*p* ≤ 0.001, <0.001, 0.001). In the within-group comparisons, the inpatient group showed significant symptom reductions at all time points postintervention (*p* ≤ 0.001, 0.014, 0.012). In the outpatient group, there was a non-significant reduction in depression at T2 (*p* = 0.246), a significant symptom reduction at T3 (*p* = 0.001) and a non-significant symptom reduction at T4 (*p* = 0.106).

There was an interaction effect of time by diagnosis [*F* (6, 93.929) = 3.184, *p* = 0.007]. None of the pairwise comparisons per time point were significant.

### HADS Total Score

There was a significant main effect of time [*F* (3, 88.699) = 14.613, *p* ≤ 0.001]. Compared to T1, there were successive reductions in HADS total scores across all time points. Whereas scores at T2–T4 were significantly lower (*p* ≤ 0.001) than scores at T1, the scores at T4 were not (*p* = 0.599). In the within-group comparisons, the inpatient group showed significant reductions at all time points postintervention (*p* ≤0.001, 0.002, 0.001). In the outpatient group, there was a non-significant reduction at T2 (*p* = 0.165) and significant reductions at T3 (*p* ≤ 0.001) and T4 (*p* = 0.007).

There was an interaction effect of time by diagnosis [*F* (6, 88.862) = 2.892, *p* = 0.013]. None of the pairwise comparisons per time point were significant.

### SF 36 Pain

There was a significant main effect of time [*F* (3, 94.503) = 20.077, *p* ≤ 0.001]. Compared to T1, there were successive reductions in pain across time points (all *p* ≤ 0.001). In the within-group comparisons, the inpatient group showed a non-significant pain reduction at T2 (*p* = 0.094) and significant pain reductions at T3 (*p* = 0.008) and T4 (*p* = 0.028). In the outpatient group, there were significant reductions in pain at T2–T4 (all p ≤ 0.001) compared to T1.

### SF 36 Pain Interference

There was a significant main effect of time [*F* (3, 103.869) = 29.784, *p* ≤ 0.001]. Compared to T1, all other time points showed significant reductions in pain interference with work (all *p* ≤ 0.001). In the within-group comparisons, both the inpatient group and outpatient group showed highly significant reductions in pain interference at T2–T4 compared to T1 (inpatient *p* ≤ 0.001, 0.001, 0.001 vs outpatient, all *p* ≤ 0.001).

### Work Ability Index

**FIGURE 2:**
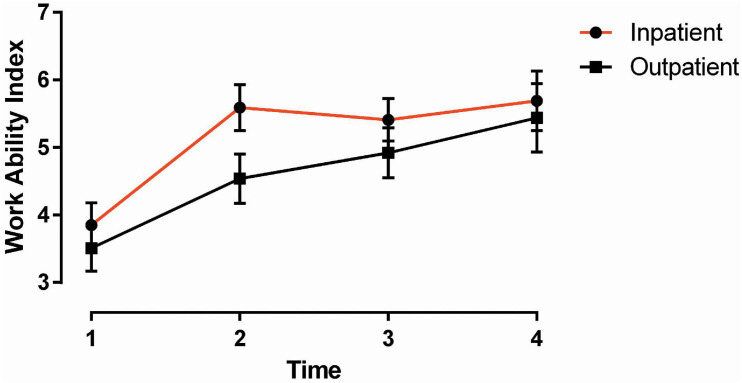
Work Ability Index: group by time. Vertical bars denote 1 S.E.M.

There was a significant main effect of time [*F* (3, 97.479) = 31.461, *p* ≤ 0.001] (Figure 2). Compared to T1, there were successive improvements in work ability at all three time points (all *p* ≤ 0.001). In the within-group comparisons, the inpatient group showed significant improvements in work ability at all time points postintervention (all *p* ≤ 0.001). The same was found in the outpatient group (*p* = 0.005, <0.001, <0.001).

### Fear-Avoidance Work

There was a significant main effect of time [*F* (3,85.91) = 6.271, *p* = 0.001]. Only fear-avoidance scores at T3 were significantly lower than scores at T1 (*p* = 0.001), whereas T2 and T4 scores were non-significant (*p* = 1.0 and 0.080). Within-group comparisons showed similar reductions in fear avoidance (FA) patterns for both groups; the inpatient group showed a significant reduction from T1 to T4 (*p* = 0.047). The outpatient group showed a significant reduction from T1 to T3 (*p* = 0.014).

The main effect of diagnosis was significant [*F* (2, 116.867) = 3.34, *p* = 0.039], showing higher FA scores in patients with musculoskeletal pain as the main referral diagnosis than in patients with mental pain as the main referral diagnosis (*p* = 0.056).

### Fear-Avoidance for Physical Activity

There was a significant interaction of Group by Time [*F* (3, 98.38) = 3.5, *p* = 0.018]. Pairwise comparisons showed a difference between groups at T4 (*p* = 0.03), with lower fear-avoidance for physical activity in the inpatient group than in the outpatient group.

In the within-group comparisons, the inpatient group showed reductions in fear-avoidance for physical activity across all time points (*p* = 0.002, <0.001, <0.001). The outpatient group showed small but non-significant reductions at T2 (*p* = 1.0) and T3 (*p* = 0.2) and an increase in fear-avoidance for physical activity from T3 to T4 (*p* = 1.0).

The main effect of diagnosis was significant [*F* (2, 104.73) = 3.33, *p* = 0.04], showing higher fear-avoidance scores in patients with musculoskeletal pain as the main referral diagnosis than in patients with mental pain as the main referral diagnosis (*p* = 0.037).

### Return to Work Self-Efficacy (RTWSE) Work Demands

There was a significant main effect of time [*F* (3, 84.837) = 12.366, *p* ≤ 0.001]. Compared to T1, there were successive improvements in RTWSE work demands at all three-time points post-intervention (all *p* ≤ 0.001). In the within-group comparisons, the inpatient group showed significant improvements in their self-efficacy for coping with work demands at all time points (all *p* ≤ 0.001). Only the T3 score showed significant improvement in the outpatient group compared to the T1 score (*p* = 0.049), whereas the two other time points showed non-significant improvements in self-efficacy for work demands (*p* = 0.220 and 0.219).

### RTWSE Modifying Tasks

There was a significant main effect of time [*F* (3, 88.426) = 6.965, *p* ≤ 0.001). Compared to T1, there were successive improvements in RTWSE Modifying job tasks at all three time points post intervention (*p* ≤ 0.001, 0.008, 0.026). In the within-group comparisons, the inpatient group showed significant improvements at all time points (*p* = 0.003, 0.022, 0.028). In the outpatient group, none of the postintervention time points were different from T1 (*p* = 0.106, 0.437, 1.000).

### RTWSE Communicating Needs

No significant results were found for RTWSE communicating needs.

### Return to Work (RTW) Expectation

There was a significant main effect of time [*F* (1, 104.621) = 16.378, *p* ≤ 0.001]. Pairwise comparisons within groups showed a significant increase in RTW expectations for both the inpatient (*p* = 0.012) and outpatient groups (*p* = 0.002).

## Discussion

Overall, the most pronounced finding was the stable and often highly significant main effect of time, showing that both rehabilitation programs improved health- and work-related outcomes. Consistent with our primary hypothesis, we found that the inpatient group improved more over time on musculoskeletal pain and fear-avoidance for physical activity than the outpatient group. However, the other group-by-time interactions were non-significant. Additionally, consistent with our main hypothesis, between-group effect sizes were of small or greater magnitude (i.e., meaningful differences in effect size) and were greatly in favor of the inpatient group. Within-group effect sizes were, on average, also in favor of the inpatient group. Two-thirds of their effect sizes were in the medium and large range, and in contrast to the outpatient group, the inpatient group displayed no zero effects and had fewer small effects. Moreover, within-group contrasts from the start until the end of the rehabilitation stay were strongest for the inpatient group. The inpatient group showed statistically significant improvements on ten out of the thirteen outcome measures at departure compared to five out of thirteen in the outpatient group. In general, the outpatient group obtained the strongest improvements at three months, thus showing a more delayed effect than the inpatient group. At 3 and 12 months postintervention, the groups generally performed at similar levels on health-related outcomes, whereas the inpatient group generally displayed stronger improvements in work-related outcomes than the outpatient group at 12 months.

### Health-Related Outcomes

Causes of sickness absence in Norway show largely the same profile as in other Scandinavian and European countries and are heavily dominated by diagnoses and complaints related to musculoskeletal pain and CMDs (Finnes et al., 2019; NAV, 2020). CMD, chronic pain, and SHC have been found to predict the frequency of medical consultations, sick leave length, and work disability (Steinsbekk et al., 2007; Brage et al., 2010; Indregard et al., 2013; Knudsen et al., 2013). Moreover, symptom severity has been found to predict RTW in both CMD (Nielsen et al., 2011) and chronic pain populations (Breivik et al., 2006). Therefore, an important aim with multidisciplinary occupational programs is to provide treatment components, such as multimodal CBT used in the present study, that might improve the management of disabling symptoms.

Among the health-related outcomes, the strongest improvements were found on symptoms related to pain as measured by the SHC (Figure 1) and the SF36. Whereas the SHC measures the severity and duration of specific musculoskeletal symptoms, the SF-36, in addition to global pain, measures pain interference with work. Both groups obtained treatment effects equaling medium magnitude in these scales. The largest improvement, however, and contrary to our hypothesis, was found in pain interference with work, in which the outpatient group obtained a large effect size on average. Regarding SHC pseudoneurological symptoms, the inpatient group showed stronger improvements than the outpatient group, obtaining average symptom improvements of medium magnitude compared to low magnitude in the outpatient group.

When assessing the clinical utility of interventions, the smallest amount an outcome must improve to be meaningful to patients is often called the minimal clinically important difference (MCID). The MCID for the SF36 pain subscales has been computed in some studies on chronic pain populations, and the results obtained in the present study are comparable to and slightly above these cutoffs, with the exception of the result on pain for the inpatient group (Lauridsen et al., 2006; Ward et al., 2014). We consider these to be strong results in a transdiagnostic population and with a relatively high comorbidity of CMDs in both groups. We found no investigative accounts in which the MCID of the SHC has been explored. Thus, we are less certain that the results obtained here reflect clinically meaningful changes for the patients. However, effect sizes were well established in the moderate range and are stronger than results from comparable studies (Johansen et al., 2016; Nottingnes et al., 2019; Gismervik et al., 2020).

Regarding anxiety and depression, we found comparable amounts of symptom reduction in both groups that were stable over time. There was a decrease in symptoms on the HADS total score, equaling roughly three points in the inpatient group and just below two points in the outpatient group. These improvements reflect MCID, which often varies between 1.5 and 2 in randomized controlled trials for various somatic patient populations (Puhan et al., 2008; Lemay et al., 2019).

### Work-Related Outcomes

We included measures of work ability, RTW self-efficacy, FA, and RTW expectations, all of which have been identified as prognostic factors for sickness absence, RTW, and other related outcomes (Oyeflaten et al., 2008; Ahlstrom et al., 2010; Aasdahl et al., 2019; Brenninkmeijer et al., 2019). These measures resemble important process variables that the rehabilitation programs in the present study aim to improve by focusing on work-related processes to facilitate RTW.

Among the work-related outcomes, the strongest improvements were obtained on work ability (Figure 2). Work ability refers to a person’s work-related functional capacity and ability to continue working in her or his current job, given the challenges and demands of the job and the person’s competence and skills (Ilmarinen, 2009). Its predictive validity for RTW-related outcomes such as sick leave and workforce departure is considered high in European samples (Ahlstrom et al., 2010; Bethge et al., 2013; Schouten et al., 2016; Lundin et al., 2017; Aasdahl et al., 2018). At departure in the present study, we found that 73% of the patients in the inpatient group improved their work ability compared to 52% in the outpatient group. Compared with three other studies on RTW, which are comparable on patient sample (i.e., transdiagnostic), method, and length of program (Braathen et al., 2007; Vindholmen et al., 2014; Johansen et al., 2019), the results from the present study are superior. In one of these three studies (Braathen et al., 2007), the vocational rehabilitation program was compared to a waitlist control group, and the results showed that work ability at both baseline and follow-up strongly predicted RTW and sickness absence. Compared to these results, one might assume that improvements obtained in the present study should be meaningful and of predictive value.

The inpatient group steadily improved across time points in FA for physical activity. Compared to baseline, there was a reduction of 3.5 points at 12 months, equaling an improvement of medium magnitude. There was a statistically significant difference between groups at 12 months, mostly because the outpatient group showed a marked increase in FA at this time point. The strongest reduction in FA in the outpatient group appeared at three months and equaled 1.7 points. Concerning MCID, we found one relevant study performed on a low back pain sample. Compared to the MCID found in this study (3.69), neither group in the present study reached this limit (Monticone et al., 2020), although the inpatient group came very close. However, the FA construct is specific for those in our sample with chronic pain. With a high referral rate on CMDs in both groups, it is natural to assume that our results on FA might be underestimated and that at least the reduction observed in the inpatient group might still reflect a clinically meaningful change.

Return to work self-efficacy and RTW expectations are conceptually similar and highlight the importance of one’s own expectations and beliefs in the ability to take necessary actions to RTW. Both concepts have shown strong predictive validity for benefit recipiency, sickness absence, and RTW in CMD and chronic pain samples (Reme et al., 2009; Shaw et al., 2011; Lovvik et al., 2014; Opsahl et al., 2016). Both groups improved on these scales with effect sizes in the low and moderate range. This demonstrates that both programs were able to improve participants’ beliefs in their current ability to resume job responsibilities and raise expectations for RTW.

### Strengths and Limitations

The major strength of this study is the manipulation of only one independent variable, namely, the length of the programs, and that programs were performed by the same team of professionals and at the same institution. This design allows for addressing a yet unexplored issue of what the optimal length of multidisciplinary occupational rehabilitation programs might be. The main limitation is the non-randomized design and lack of a passive control group (i.e., waitlist/natural history) for a more appropriate calculation of treatment effects than baseline-posttest comparisons within groups. As selection to the programs was predetermined by the external employer (NLWA), there were some differences at baseline, which we accounted for in the statistical model. Furthermore, effect sizes were computed on unadjusted scores for simplicity of interpretation. Between-group differences in effect sizes should thus be interpreted with caution.

## Concluding Remarks

The present study demonstrates that multidisciplinary rehabilitation based on multimodal CBT for complex disorders is efficacious in improving self-rated health and work ability, and other work-related outcomes. Despite their different lengths, both programs produced significant improvements at levels that in other comparable studies have been demonstrated as clinically meaningful or with predictive validity for RTW and sickness absence. Consistent with our hypothesis, the inpatient group improved more overtime on musculoskeletal pain and fear-avoidance for physical activity than the outpatient group. Within- and between-group effect sizes were also slightly in favor of the inpatient group, particularly at the end of rehabilitation and at twelve months for work-related outcomes. Moreover, the overall amount of improvements demonstrates two programs with clinical utility in helping a patient population that in general is considered hard to treat and where treatment effects on health outcomes are usually low or non-existent (Kamper et al., 2015; Aasdahl et al., 2017; Finnes et al., 2019; Gismervik et al., 2020). As such, the results from the present study stand out as consistent and clinically meaningful in most analyses. Ideally, an effective intervention should reduce sickness absence and improve health, but the literature often shows that sickness absence is improved without improvements in health or the opposite (Finnes et al., 2019). However, one should assume that for improvements in health and work-related outcomes to be predictive of sickness absence, the improvements have to be clinically meaningful. However, in this trial, it remains to be seen whether the positive improvements are predictive of sickness absence and RTW and whether the inpatient program proves to be cost-effective.

## Data Availability Statement

The dataset generated and analyzed during the current study is available from the corresponding author on reasonable request.

## Ethics Statement

The studies involving human participants were reviewed and approved by South-East Regional Committee for Medical and Health Research Ethics, Norway (2013/1559). The patients/participants provided their written informed consent to participate in this study.

## Author Contributions

PSL and TJ contributed to the study conception and design. TJ was responsible for data collection. PSL and PA performed the statistical analysis. PSL wrote the first draft of the manuscript, and all authors commented on and reviewed later versions of the manuscript. All authors read and approved the final manuscript.

## Conflict of Interest

The authors declare that the research was conducted in the absence of any commercial or financial relationships that could be construed as a potential conflict of interest.

## Publisher’s Note

All claims expressed in this article are solely those of the authors and do not necessarily represent those of their affiliated organizations, or those of the publisher, the editors and the reviewers. Any product that may be evaluated in this article, or claim that may be made by its manufacturer, is not guaranteed or endorsed by the publisher.

## Abbreviations

MRP: multidisciplinary rehabilitation program
RTW: return to work
CMD: common mental disorders
SHC: subjective health complaints
CBT: cognitive behavior therapy
RTWSE: return to work self-efficacy
HADS: the hospital anxiety and depression scale
FA: fear avoidance
NLWA: the Norwegian labor and welfare administration
LMM: linear mixed models
ES: effect size
MCID: minimal clinically important difference.
